# Bubble Rupture and Bursting Velocity of Complex Fluids

**DOI:** 10.1021/acs.langmuir.2c01875

**Published:** 2022-10-26

**Authors:** Nicola Antonio Di Spirito, Shadi Mirzaagha, Ernesto Di Maio, Nino Grizzuti, Rossana Pasquino

**Affiliations:** DICMaPI, Università degli Studi di Napoli Federico II, P.le Tecchio 80, 80125 Napoli, Italy

## Abstract

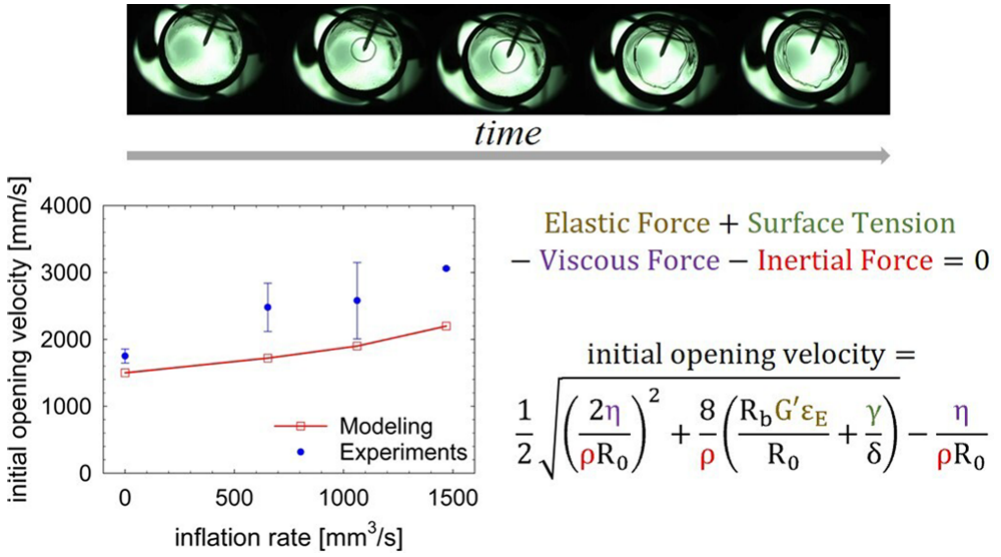

We analyzed bubble rupture and hole opening dynamics in a non-Newtonian fluid by investigating the retraction process of thin films after inflation at different blowing rates. The experiments were modeled through a dimensional analysis, with the aim of establishing a general approach on the bubble rupture dynamics and discerning the role of viscous, elastic, surface, and inertial forces on the opening velocity, according to the nature of the specific fluid. A new mathematical model, which includes all possible contributions to the hole opening dynamics, was proposed, to the best of our knowledge for the first time. The experimental evidence on the opening velocity as a function of the inflation rate was found to be in good agreement with the prediction of the model. The sensitivity of our modeling was tested by comparing our results with the existing models of retracting velocity.

## Introduction

Bubble rupture and liquid sheet retraction are of paramount importance in many scientific and technological fields and areas of daily life,^1^ such as aerosol formation,^2−5^ bubbly magma rheology,^6−9^ industrial and food foam processes,^10^ and biology and drug delivery.

The first studies on the rupture of bubbles were performed in the second half of 1800 by Dupré^11^ and Rayleigh,^12^ who observed soap bubbles during their bursting. Later, Ranz^13^ presented an experimental work about soap films and their bursting after a puncture, describing the role of surface tension during film retraction and found that the retracting velocity was nearly constant. A decade later, Taylor^14^ and Culick^15^ tried to apply a theoretical approach to Dupré derivations.^11^ Specifically, they inferred a mathematical relation for the film retracting velocity *U*
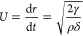
1where d*r*/d*t* is the variation of the hole radius with respect to time, γ the surface tension between the liquid and the surrounding gas, ρ the density of the liquid, and δ the thickness of the film. Taylor and Culick’s study was subsequently corroborated by McEntee and Mysels,^16^ who experimentally studied the bursting of soap films. Keller,^17^ years later, theoretically analyzed the rupture of nonuniform liquid sheets. The previous studies involved inviscid liquid films, such as water sheets or soap films in air. The first study on viscous fluids was performed by Debrégeas et al.,^18,19^ who investigated the effects of viscous contribution when inertia can be considered negligible. They found that, because of the high viscosity of the fluid, the film retracting velocity is slower than in the inviscid case, and is not a constant, in contrast with the prediction of eq 1. Debrégeas et al.^18,19^ proposed the following time-evolving growth law for the hole radius, *r*(*t*):
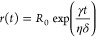
2where *R*_0_ and η are the initial hole radius and the fluid viscosity, respectively. The initial retracting velocity of the film, v_0_^V^, is therefore
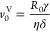
3

Evers et al.^20^ studied the retraction process of very thin viscoelastic films initially at rest, observing a retraction velocity much slower than that of Newtonian films because of the intrinsic film elasticity. Brenner and Gueyffier^21^ focused on retraction phenomena and rim formation of very viscous films, demonstrating the importance of viscous, surface tension, and inertial contributions. Brenner and Gueyffier’s analysis was enriched with numerical investigations by Song and Tryggvason,^22^ who considered the effect of an ambient fluid around the film, and then by Sünderhauf et al.,^23^ who focused on the presence of inertia or viscosity. Dalnoki-Veress et al.^24^ investigated the formation and growth of holes in polymer films. Bubble rupture at a free surface was numerically studied by Duchemin et al.,^25^ who used direct numerical simulations based on the Navier–Stokes equations. In 2009, Savva and Bush^26^ presented an analysis on the retraction of planar and circular liquid sheets, highlighting the role of the viscosity contribution, geometry, and initial conditions. Later, Villone et al.^27,28^ used numerical simulations to investigate the retracting process of viscoelastic films. Sabadini et al.^29^ experimentally investigated the effects of the elastic contribution in soap bubbles. Then, Tammaro et al.^30^ gave a complete explanation of the role of elasticity, showing that, besides the properties of the fluid and the geometry of the bubble, the inflation process and the deformation history must be considered. They found a mathematical expression of the initial retracting velocity, v_0_^E^, for a viscoelastic bubble, which accounts for both viscous forces and elasticity and neglects inertia:
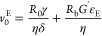
4where *R*_b_ represents the initial radius of the bubble, *G*′ is the fluid elastic modulus, and ε_E_ is the recoverable deformation. Equation 4 was obtained from a force balance that includes surface tension and elastic contributions, which promote hole bursting, and the viscous contribution, which, on the other hand, slows down the opening process. It shows the dependence of the initial retracting velocity of a viscoelastic fluid on the inflation rate and on the initial bubble radius. Walls et al.^31^ analyzed the role of gravity and the viscous contribution in jet drops. Furthermore, bubble rupture and droplet ejection were experimentally investigated by Ghabache and Séon,^32^ who examined the size of the top jet drop after the bursting of a bubble. The formation of a liquid jet in bubble bursting, with consequent formation of emitted droplets, was studied by Ganan-Calvo,^33^ who proposed a scaling law of the droplet size and jet velocity. Lai et al.^34^ published a study on the dynamics of bubble rupture, describing the collapse of the cavity and the production of a jet, considering the influence of capillary inertia and viscous forces. The minimum size of the drops ejected from the rupture of a bubble was investigated by Brasz et al.,^35^ who adopted high-speed camera visualization and numerical calculation. Deike et al.^36^ focused on the jet velocity by means of experiments and simulations. Botta et al.^37^ conducted experimental studies on soap bubble rupture, recording the phenomena of bursting by means of a high-speed camera and experimentally identifying the parameters of the materials. Then, Sen et al.^38^ investigated the effects of viscoelasticity in inkjet printing to avoid satellite droplet formation. Gañán–Calvo and López–Herrera^39^ analyzed bubble bursting, proposing the prediction of the size and speed of ejected droplets. Tammaro et al.^40^ analyzed the role of surface viscoelasticity in bubble rupture, illustrating the circumstances under which bubbles exhibit a flowering-like morphology during bursting. Sanjay et al.^41^ numerically studied the formation of capillary waves of bursting bubbles in a viscoplastic medium. Very recently, the impact of material properties, such as surface tension, viscosity, and density, on the production of gas jets from bubble bursting was examined by Dasouqi et al.^42^

Understanding the bubble rupture phenomena and the corresponding complex fluid dynamics proves to be nontrivial because of the presence of different forces acting on the system. It is not a case, indeed, that eqs 1, 3, and 4 refer to the same quantity, which is the retracting velocity, although named with different symbologies to mark specific predominances. A good approach to tackle such problems to a significant extent is the use of dimensional analysis,^43^ in a way to fully understand the forces at play and their relative magnitude. The introduction of specific dimensionless groups is particularly useful in understanding the behavior of complex fluids. To this end, three important dimensionless parameters may be considered:^44^ Reynolds number, *Re* = ρ*v*δ/η, capillary number, *Ca* = η*v*/γ, and Weissenberg number, *Wi* = λ*v*/δ, where v represents a characteristic velocity, δ is a characteristic length, and λ stands for the relaxation time of the fluid. *Re* allows for a comparison between inertial and viscous forces. *Ca* represents the relative effect of viscous to surface forces. *Wi* compares elastic and viscous forces. The ratio *Wi*/*Re* defines the elasticity number,^45^*El* = ηλ/ρδ^2^ – that is, the comparison between inertial contributions and elasticity. The relation *Re*/*Ca* results in the inverse of the squared Ohnesorge number, *Oh*^–2^ = ργδ/η^2^.^46^ Elastic and capillary effects vs viscous contributions can be described by the elasto-capillary number,^47^*E*_c_ = *Wi*/*Ca* = λγ/ηδ. Depending on the fluid properties and flow conditions, the phenomenon can be described based on the magnitude of these significant dimensionless parameters.

In this paper, bubble rupture and retraction phenomena were investigated experimentally on a non-Newtonian, viscoelastic fluid, suitably chosen for its rheological properties. Furthermore, the balance of different forces governing the system was studied, and a generalized approach based on dimensional analysis was proposed. Specifically, we used a Carbopol solution as the test material since it represents an ideal candidate to test the effectiveness of dimensional analysis when all forces at play must be contemplated. Film retracting velocities following bubble rupture were experimentally determined and compared with those evaluated by using a novel theoretical equation derived from dimensional analysis. To assess the sensitivity of our mathematical model, we compared it with existing models of retracting velocity.

## Materials and Methods

### Materials

A water-based solution of an acrylic acid-based polymer — Carbopol Ultrez 10 (Lubrizol Co., Wickliffe, OH) — was used to perform bubble rupture experiments. The polymer was dispersed in demineralized water at room temperature and mechanically stirred to guarantee a homogeneous distribution.^48^ The rheology of the solution is strongly dependent on polymer concentration and pH.^49,50^ In this case, a 0.10 wt % Carbopol solution at pH 8.30 was used.

### Rheological Characterization

The rheological properties of the fluid were experimentally studied by a stress-controlled rheometer (MCR702; Anton Paar GmbH, Graz, Austria), equipped with cone-plate geometries (CP 50-1: diameter 50 mm and cone angle 1° and CP 25-1: diameter 25 mm and cone angle 1°). Flow curve tests were performed in a range of shear rates between 100 and 10^–5^ s^–1^, at 25 °C. Moreover, the sample viscoelasticity was studied by frequency sweep tests at 25 °C — in an angular frequency range between 100 and 0.10 rad/s — both at strains low enough to guarantee the linear viscoelastic regime and at a stress value able to simulate the rheological behavior of the fluid, once punctured.

### Bubble Rupture Analysis

**Figure 1:**
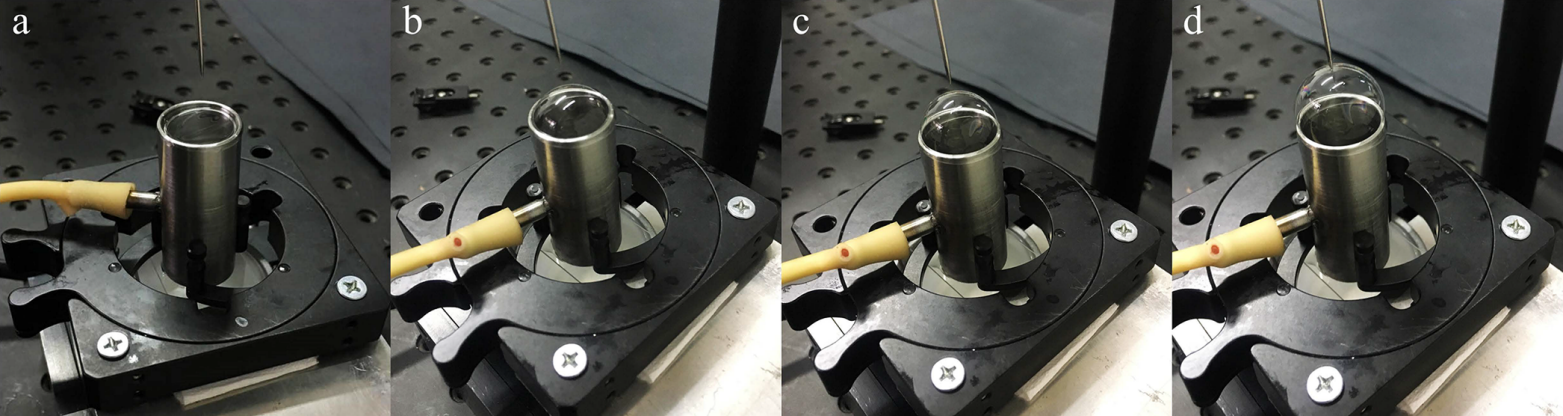
Blowing process steps: (a) film formation; (b) start of bubble inflation; (c) growth of the bubble; (d) bubble collision against the needle.

The investigation of the bubble rupture process was conducted considering a homemade apparatus and blowing protocol as used by Tammaro et al.^30^ to visualize the bubble rupture and measure the retracting velocity of the hole opening. Thin flat films of fluids were deposited on a metallic cylinder with a radius of 9 mm. The film was inflated by air injected from a syringe pump (Model 22 syringe pump; Harvard Apparatus, Holliston, MA) through a silicone tube, at different flow rates *Q*. A needle with a tip with a radius of 62 μm was placed on top of the blown film, to puncture and break the bubble. A high-speed camera capable of acquiring up to 10^5^ frames/s (i-speed 3; Olympus Scientific Solutions, Waltham, MA) was used during bubble bursting analysis to record the bubble rupture and the subsequent film retraction stage (with a frame rate of 1.5 × 10^4^ frames/s). Optimal light conditions, required for high-speed recording, were guaranteed by a 75 W LED lamp placed behind the film, chosen so that the light source did not significantly heat the film by radiation. In Figure 1a–d, the blowing process steps, from film formation to bubble collision against the needle, are shown.

**Figure 2:**
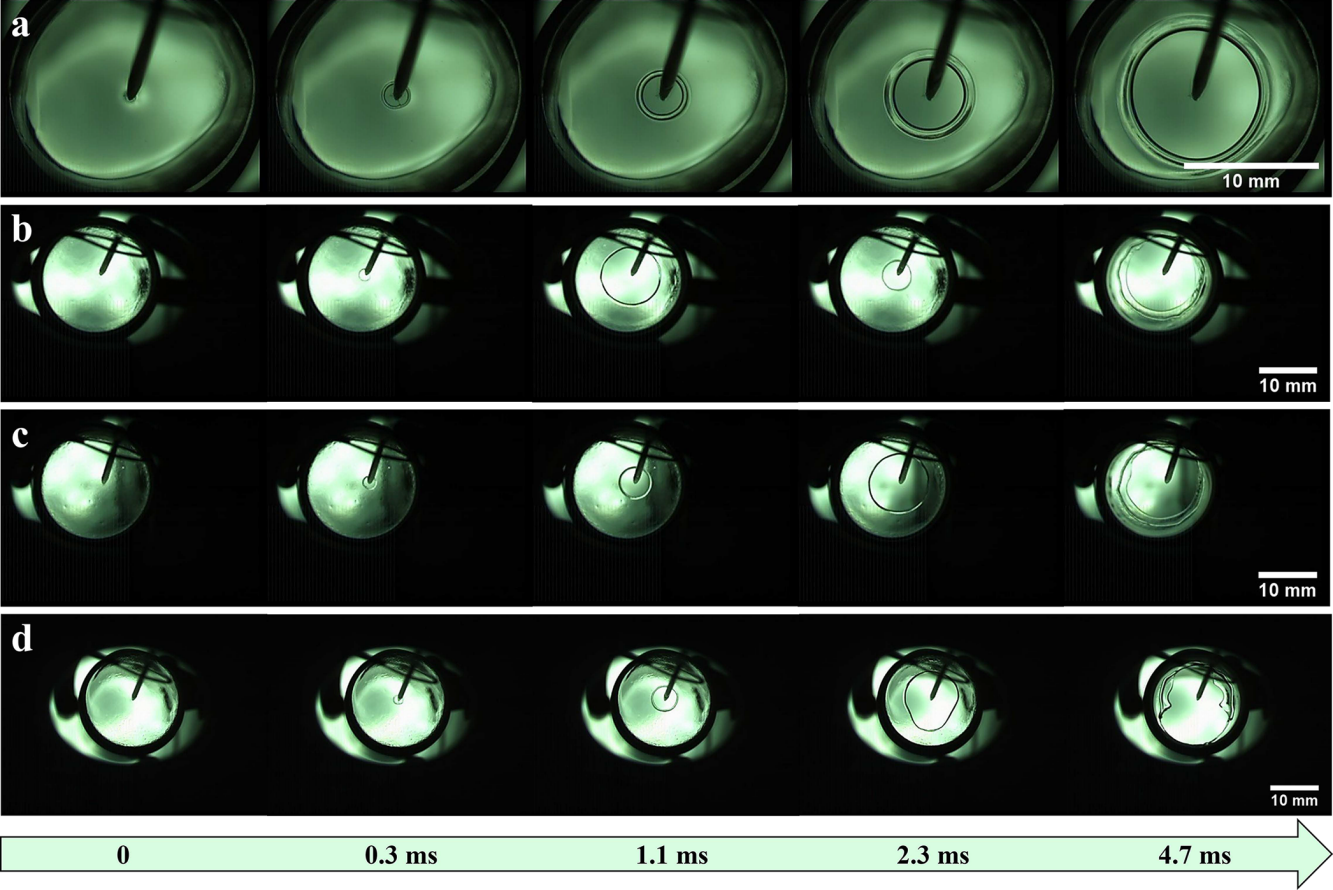
Experimental images of the bubble opening sequence for the 0.10 wt % Carbopol solution at different *Q* values: (a) *Q* = 0; (b) *Q* = 653 mm^3^/s; (c) *Q* = 1062 mm^3^/s; (d) *Q* = 1470 mm^3^/s. The timestamps of the snapshots are shown in the green arrow.

The acquired digital frames were analyzed using ImageJ free software to measure the hole radius during film retraction as a function of time, *r*(*t*). From these data, the initial hole opening retracting velocity, v_0_, was computed considering the very early times of the radius vs time plot. As an example, Figure 2a–d shows the acquired images of the opening films of the 0.10 wt % Carbopol solution during the inflation process at different inflation rates.

Figure 2 reveals the presence of a jagged profile of the rim, which is more apparent when the inflation rate is increased. These fractured shapes are probably explained by a high *E*_c_ and a high deformation rate. Under such conditions, cracks on the opening liquid film appear in the final stages, as already reported by Tammaro et al.^40^ However, this morphological profile does not affect the hole opening and the film retracting velocity during the initial bubble bursting times.

**Figure 3:**
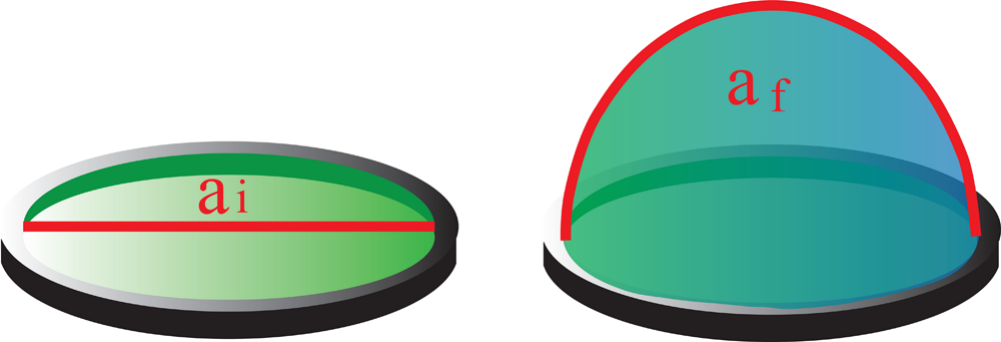
Sketch of the time evolution of the bubble arc.

A standard secondary webcam (HD Pro C920; Logitech Europe S.A., Lausanne, Switzerland) was used for macroscopic observation of the process, to measure the recoverable deformation, ε_E_, imposed by the blowing apparatus on the liquid film. In particular, bubble growth during inflation was measured, and the variation of the bubble arc over time was calculated using ImageJ software. ε_E_ was estimated from the total applied deformation, ε_T_, as ε_E_ ≈ ε_T_ exp (−τ_i_/λ),^30^ where τ_i_ and λ are the inflation time and the fluid relaxation time, respectively. ε_T_ can be experimentally measured as (a_f_ – a_i_)/a_i_,^30^ where a_f_ represents the arc measured at τ_i_ and a_i_ corresponds to the diameter of the flat film before the start of the blowing process (Figure 3).

The method adopted to compute the thickness of the liquid films, δ, was based on measuring the weight of the amount of liquid at bubble rupture. After the deposition of the fluid on the metallic cylinder and the inflation procedure, the bubble was formed, and its breakage was performed against a sheet of paper. The fluid ring was weighed, and its thickness was computed as δ = *m*/(ρ*A*), where *m* and *A* are its mass and its “printed” area, respectively. δ was assumed to be uniform.

All experiments were conducted at room temperature.

## Results and Discussion

### Rheology

**Figure 4:**
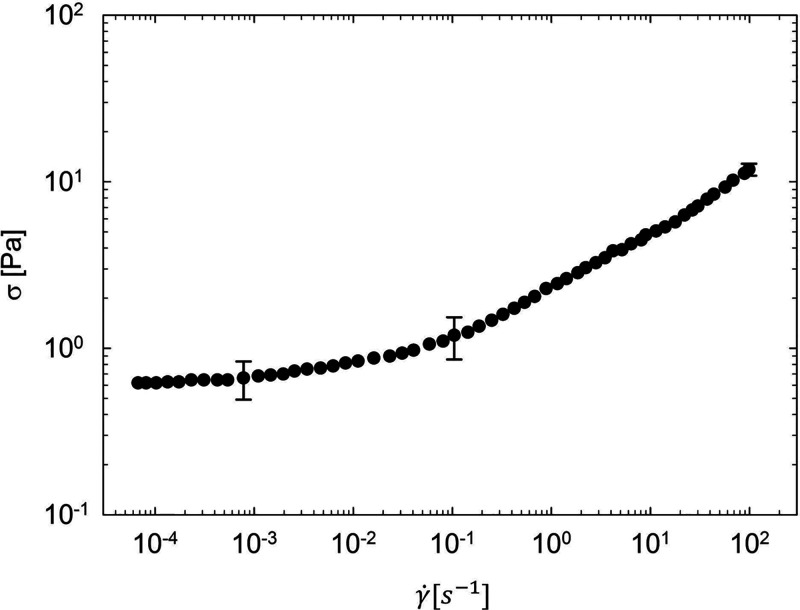
Flow curve of the Carbopol sample at 25 °C. The error bars are evaluated as the standard deviation of multiple experiments.

**Figure 5:**
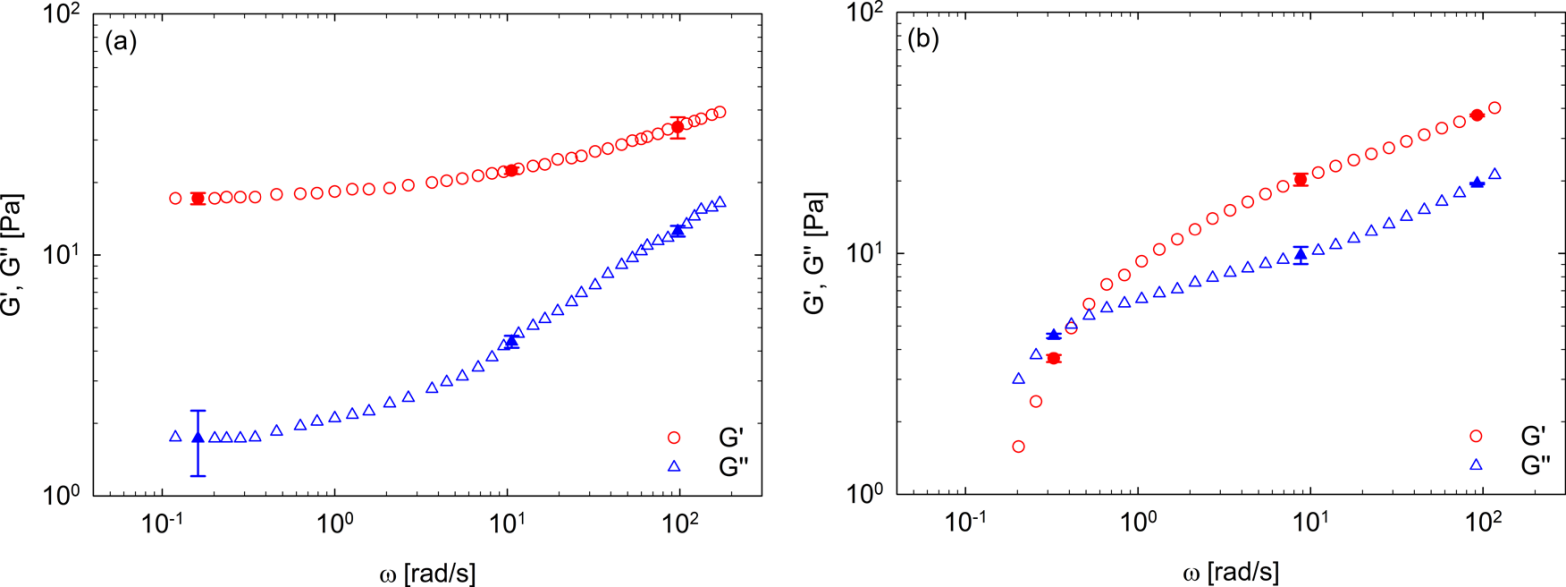
Linear viscoelastic behaviors of the Carbopol sample: (a) before yielding (0.1% strain); (b) after yielding (stress of 1 Pa). The error bars are evaluated as the standard deviation of multiple experiments.

The flow curve of the Carbopol sample, relating the shear stress (σ) to the shear rate (γ̇), is represented in Figure 4. The viscosity of the fluid decreases with increasing shear rate, showing a shear thinning behavior when a characteristic yield stress value is overcome. The curve was fitted using the Herschel–Bulkley model (eq 5)
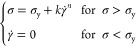
5where *k* and *n* are the consistency and flow indices, respectively, and σ_y_ is the yield stress. Specifically, *k*, *n*, and σ_y_ are equal to 1.6 ± 0.1 Pa·s*^n^*, 0.42 ± 0.01, and 0.58 ± 0.05 Pa, respectively. The rheological behavior of the Carbopol solution was further characterized by frequency sweep tests. Figure 5a depicts the viscoelastic moduli of the sample, *G*′ and *G*″, at 25 °C and low strain, before yielding. The system shows solid-like behavior in the whole frequency range, as suggested by the storage modulus *G*′ being larger than the loss modulus *G*″. Linear viscoelasticity consolidates the existence of a yield stress, as shown by the *G*′ plateau at low frequencies.^51^

A frequency sweep test was also performed by imposing a stress value equal to 1 Pa. This value of stress is an estimation of the stress felt by the fluid during the blowing process, evaluated by taking into consideration the measured deformation rate (i.e., the total deformation over the inflation time) and the fluid constitutive equation (i.e., by approximating the measured deformation rate with the shear rate). More specifically, Figure 5b reports *G*′ and *G*″ on the yielded sample at a stress of 1 Pa, higher than the yield stress value. The rheological response reported in Figure 5b is distinctive of a viscoelastic polymer network, with a well-defined crossover angular frequency (i.e., a long relaxation time) and a marked elasticity.^52^ These are the rheological characteristics of the sample that is punched by the needle after the blowing process.

### Bubble Rupture

**Figure 6:**
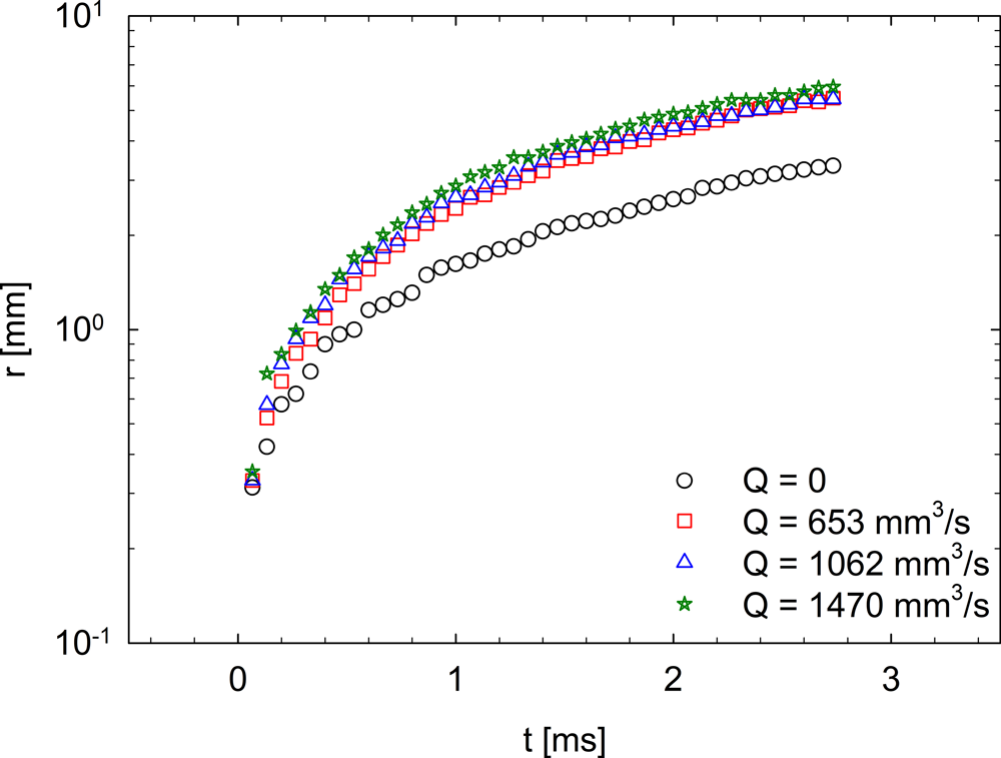
Experimental trends of the hole radius *r*(*t*) at different *Q* values for the Carbopol solution.

The transient hole radius, *r*(*t*), after bubble rupture was investigated experimentally. During the bubble formation process, different values of the inflation rate, *Q*, were considered. Figure 6 shows *r*(*t*) under static conditions (no inflation, *Q* = 0) and at different inflation rates for the Carbopol/water mixture.

**Figure 7:**
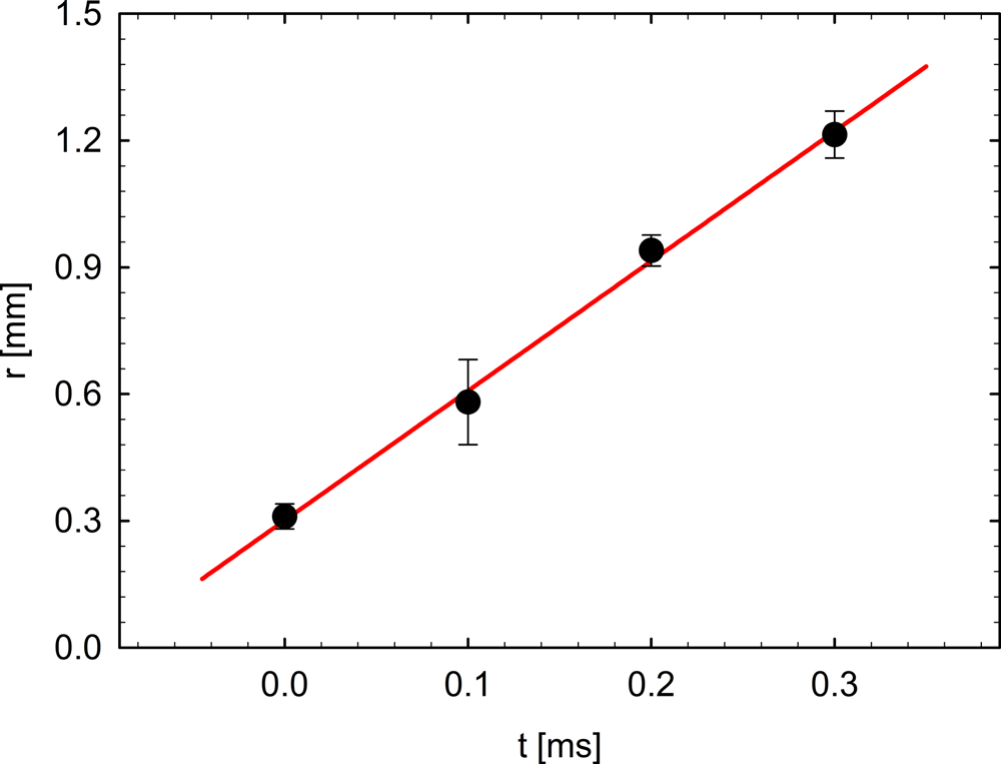
Experimental trend of the hole radius *r*(*t*) at *Q* = 1470 mm^3^/s (data from Figure 6 in the early stages). The slope of the red line gives the value of the initial retracting velocity. The error bars are evaluated as the standard deviation of multiple experiments on the same flow rate.

In particular, v_0_ was evaluated by a linear regression of the hole radius at the early times. As an example, Figure 7 illustrates the fit at *Q* = 1470 mm^3^/s for our tested solution.

Table 1 lists the mean values of the resulting initial retracting velocities, v_0_, which were computed at the beginning of the phenomenon, at different inflation rates, along with the estimated uncertainty.

**Table 1:** Experimental Values of the Initial Retracting Velocity for the Carbopol Solution at Different Inflation Rates

inflation rate, *Q* [mm^3^/s]	experimental initial retracting velocity, v_0_ [mm/s]
0	1.7 × 10^3^ ± 1.1 × 10^2^
653	2.5 × 10^3^ ± 3.6 × 10^2^
1062	2.6 × 10^3^ ± 5.7 × 10^2^
1470	3.1 × 10^3^ ± 1.7 × 10

### Modeling

Bubble rupture dynamics is governed by the presence of various forces acting on the system that, therefore, must be considered when computing the bursting velocity. In general, assuming the gravity force as negligible, the retracting velocity of the film after the bubble rupture depends on different acting contributions: the elastic force, *F*_E_ = 4πδ*R*_b_*G*′ε_E_, the viscous force, *F*_V_ = −4πδv_0_η, the inertial force, *F*_I_ = −2πδ*R*_0_ρv_0_^2^, and the surface tension, *F*_γ_ = 4πR_0_γ. While *F*_E_ and *F*_γ_ promote film retraction (both with a positive sign), *F*_V_ and *F*_I_ slow down hole opening (both with a negative sign). A force balance can be written as follows:
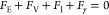
6

Table 2 represents an identity card of our investigated fluid, giving a summary of the material properties that were considered to compute the dimensionless parameters discussed previously, at different inflation rates. In addition, it reports the recoverable deformation ε_E_, measured at different *Q* values (obviously, without inflation, ε_E_ = 0). To estimate ε_E_, when *Q* ≠ 0, the fluid relaxation time has been evaluated as the inverse of the angular frequency at which the crossover between *G*′ and *G*″ appears (see Figure 5b), i.e., λ ≅ 2.5 s. Furthermore, the inflation time, τ_i_, decreases with increasing inflation rate (τ_i_ ≅ 3 s, at *Q* = 653 mm^3^/s; τ_i_ ≅ 2 s, at *Q* = 1062 mm^3^/s; τ_i_ ≅ 1 s, at *Q* = 1470 mm^3^/s). The corresponding values of *Re*, *Ca*, *Wi, Oh*^*–2*^, *El*, and *E*_c_ are shown in Table 3. For *Q* = 0, the sample is considered unyielded with a relaxation time ideally infinite.

**Table 2:** Properties of the Carbopol Solution, at Different Inflation Rates

inflation rate, *Q* [mm^3^/s]	characteristic velocity, v_0_ [mm/s]	characteristic shear rate, γ̇ [s^–1^]	viscosity, η [Pa·s]	deformation, ε_E_ [dimensionless]
0	1.7 × 10^3^	3.4 × 10^4^	3.8 × 10^–3^	0
653	2.5 × 10^3^	4.8 × 10^4^	3.1 × 10^–3^	0.11
1062	2.6 × 10^3^	5.0 × 10^4^	3.0 × 10^–3^	0.17
1470	3.1 × 10^3^	5.9 × 10^4^	2.7 × 10^–3^	0.37

**Table 3:** Dimensionless Numbers for the Carbopol Solution, at Different Inflation Rates^a^

inflation rate, *Q* [mm^3^/s]	*Re*	*Ca*	*Wi*	*Oh*^–2^	*El*	*E*_c_
0	24	0.10		232		
653	42	0.12	1.2 × 10^5^	348	2.8 × 10^3^	9.9 × 10^5^
1062	45	0.12	1.3 × 10^5^	364	2.7 × 10^3^	1.0 × 10^6^
1470	59	0.13	1.5 × 10^5^	444	2.5 × 10^3^	1.1 × 10^6^

a For *Q* = 0, *Wi*, El, *E*_c_ cannot be evaluated because of the ideally infinite relaxation time of the unyielded sample.

The fluid density, ρ, was always assumed to be equal to that of water. The characteristic length, δ, is the thickness of the fluid film, and it was found to be roughly equal to 0.052 ± 0.017 mm (variance coming from multiple measurements). The surface tension can be estimated to be ∼0.063 N/m.^53^ The characteristic velocity present in the dimensionless groups was taken as the measured initial retracting velocity of the film, v_0_, at different values of the inflation rate. The viscosity, η, is extrapolated using the flow curve at a shear rate value equal to the characteristic shear rate of the process, γ̇ = v_0_/δ. The magnitudes of the computed dimensionless numbers presented in Table 3 suggest that there is an interplay of forces that must be considered, for all the inflation rates. As a result, the whole force balance (eq 6) can be made explicit as follows:
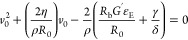
7

The positive root of eq 7 gives an expression for the initial retracting velocity as follows:
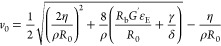
8

Equation 8 represents the retracting velocity where all forces of eq 6 are considered. It can be used to compute the initial retracting velocity for the Carbopol solution at different *Q* values. While the viscosity enters the model in the viscous retraction term, requiring the use of the value corresponding to the estimated retraction velocity gradient, the choice of the elastic modulus value is based on different grounds. In fact, the elastic modulus was taken at the frequency that corresponds to the tan φ = *G*″/*G*′ minimum (φ is the phase angle), i.e., tan φ_min_ (see the moduli in Figure 5b), equal to 23 Pa for all inflation rates. Such a choice is supported by two considerations. On the one hand, it is known that for entangled polymer systems, the value of *G*′ at tan φ_min_ is a good estimate of the plateau modulus of the material.^54^ On the other hand, the elastic term in eq 8 comes from the “loading of the spring”, corresponding to the elastic component of the polymer during the inflation stages, preceding the bubble opening. Consequently, the elasticity must correspond to the conditions applied during inflation, where the stress felt by the sample is about 1 Pa (see above), and not during the opening process.

As mentioned above, a metallic cylinder with a radius of 9 mm was used for the experiments. Therefore, the initial radius of the bubble, *R*_b_, can be considered equal to about 9 mm. Furthermore, the initial radius of the hole, *R*_0_, is equal to 62 μm, that is, the radius of the tip of the needle. It is worth noting that, if the inflation rate is 0 (null deformation), *F*_E_ = 0.

Using the parameter values defined above, the numerical derivations of the initial retracting velocity were found using eq 8 and are displayed in Table 4.

**Table 4:** Numerical Values of the Initial Retracting Velocity for the Carbopol Solution, at Different Inflation Rates

inflation rate, *Q* [mm^3^/s]	numerical initial retracting velocity, v_0_ [mm/s]
0	1.5 × 10^3^
653	1.7 × 10^3^
1062	1.9 × 10^3^
1470	2.2 × 10^3^

The retracting velocity for the Carbopol solution is, of course, not dependent on the yield stress, being the puncture performed on a yielded sample characterized by the rheological response in Figure 5b. Furthermore, the ratio that describes the comparison between the yield stress and the capillary pressure is much smaller than 1 (σ_y_δ/γ ≪ 1).

**Figure 8:**
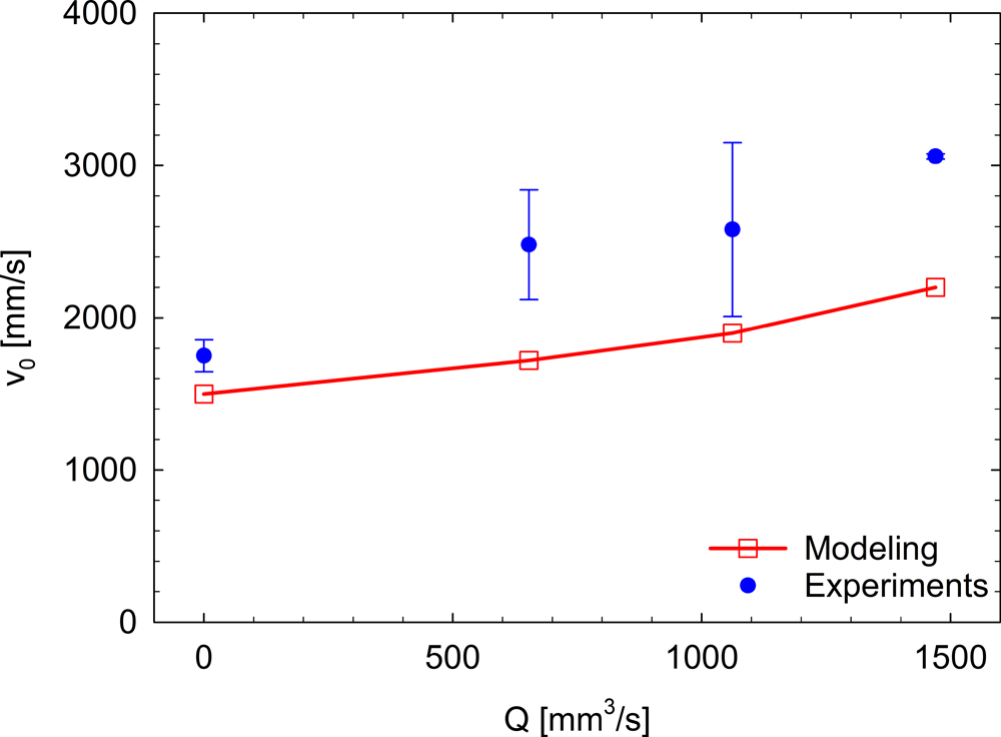
Initial retracting velocity as a function of the inflation rate. The error bars are evaluated as the standard deviation of multiple experiments with the same flow rate.

The results shown in Table 4 agree well with the experimental outcomes presented in Table 1. The newly derived eq 8 provides a mathematical description of the initial retracting velocity for a viscoelastic fluid, where, in addition to surface tension, inertia is not negligible. It represents an effective means of calculating the initial retracting velocity of a fluid film when the magnitudes of the acting forces are comparable with each other. The initial retracting velocity values found from the experiments and those derived from the mathematical model are compared in Figure 8, as a function of the inflation rate. Although the comparison is not perfect, the model, with no fitted parameters involved, reasonably well predicts the trend of the initial retracting velocity with the flow rate.

### Comparison with Existing Models

**Figure 9:**
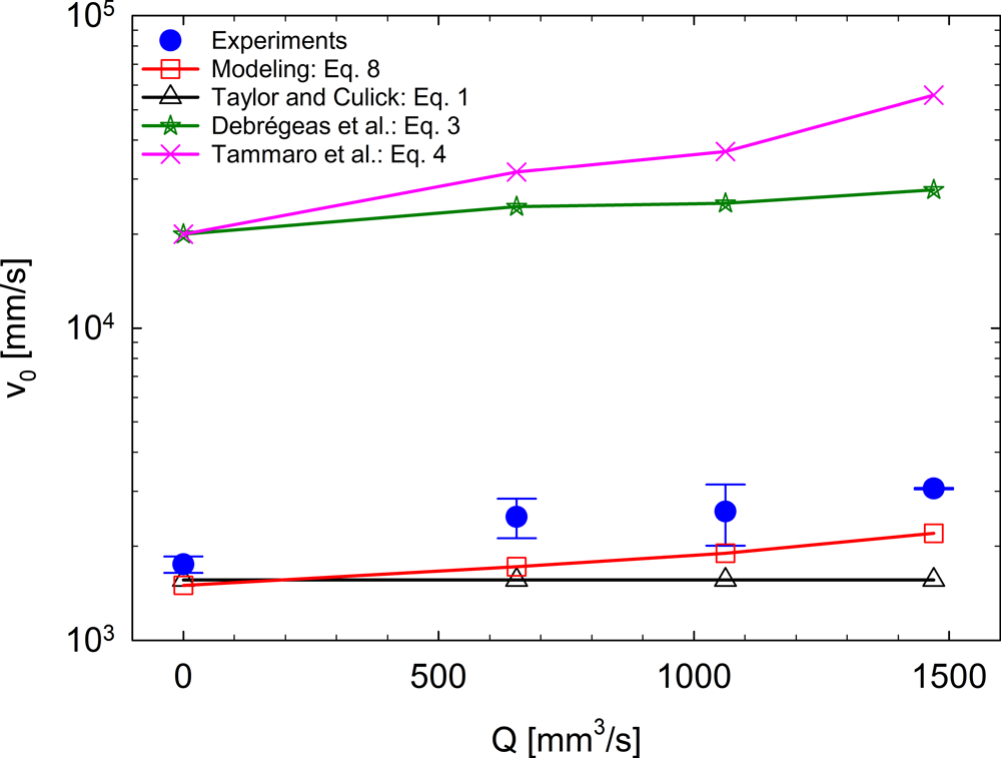
Comparison of our experimental and modeling results with predictions from existing models.

The comparison between eq 8 and the existing models of the retracting velocity is particularly useful, with the aim of testing the sensitivity of our mathematical model. In Figure 9, we report the trend of v_0_(*Q*) of our model compared to the models of Taylor and Culick,^14,15^ Debrégeas et al.,^18,19^ and Tammaro et al.^30^ Moreover, these predictions are compared with the values of the initial retracting velocities found from the current experiments. It is undeniable that our velocity prediction is the closest to the experimental data, if compared to the other existing models.

The results of this comparison are reported in Table 5.

**Table 5:** Values of v_0_(*Q*) from Experiments and through eqs 1, 3, 4, and 8

*Q* [mm^3^/s]	experiments, v_0_ [mm/s]	modeling, v_0_ [mm/s]	Taylor and Culick, v_0_ [mm/s]	Debrégeas et al., v_0_ [mm/s]	Tammaro et al., v_0_ [mm/s]
0	1.7 × 10^3^ ± 1.1 × 10^2^	1.5 × 10^3^	1.6 × 10^3^	2.0 × 10^4^	2.0 × 10^4^
653	2.5 × 10^3^ ± 3.6 × 10^2^	1.7 × 10^3^	1.6 × 10^3^	2.4 × 10^4^	3.2 × 10^4^
1062	2.6 × 10^3^ ± 5.7 × 10^2^	1.9 × 10^3^	1.6 × 10^3^	2.5 × 10^4^	3.7 × 10^4^
1470	3.1 × 10^3^ ± 1.7 × 10^2^	2.2 × 10^3^	1.6 × 10^3^	2.8 × 10^4^	5.6 × 10^4^

As previously said, eq 1 of Taylor and Culick comes from a force balance on inviscid liquid films, where the elastic and viscous forces are neglected. Equation 3 of Debrégeas et al. refers to viscous fluids and takes into account the surface tension and viscous contributions, neglecting *F*_E_ and *F*_I_. Instead, the Tammaro et al. equation (eq 4) for viscoelastic liquids is the result of neglecting only the inertial force. Clearly, the contribution of all terms in eq 8 is essential to achieve the outcomes of Figure 9 and Table 5. In fact, v_0_ computed using eqs 1, 3, and 4 are markedly different from our v_0_.

## Conclusions

Understanding bubble rupture dynamics has an overwhelming importance in many scientific and technological areas. In this paper, we analyzed complex bubble bursting and film retraction phenomena, thanks to a homemade setup and a fast camera.

The experimental investigation involved a non-Newtonian fluid characterized by a peculiar rheology. The rupture dynamics was studied experimentally, by inflating the fluid with different flow rates, and the results were modeled using a dimensionless approach. We derived a novel predictive equation for the computation of the initial retracting velocity, accounting for the elastic force, the viscous force, the inertial force, and the surface tension, and we found good agreement between the experimental outcomes and the mathematical derivations. To test the sensitivity of our mathematical model, we compared it with the existing models of the retracting velocity, which differ from the current one because they do not contemplate one or more of the forces at play. These models predict a velocity that is an order of magnitude higher than the measured one in some cases. The strength of these findings lies in the possibility of characterizing the bubble rupture dynamics of a specific fluid when all the acting forces must be contemplated.

## Supporting Information Available

Figure S1: comparison of three measurements of *r*(*t*) at *Q* = 1062 mm^3^/s for the 0.10 wt % Carbopol solution (PDF)
