# Supplementary material for: Bubble Rupture and Bursting Velocity of Complex Fluids
Source: Langmuir. 2022 Oct 26;38(44):13429–36. doi: 10.1021/acs.langmuir.2c01875 (PMC9648340; doi:10.1021/acs.langmuir.2c01875)
Supplement: Supplementary file 1 — la2c01875_si_001.pdf [file la2c01875_si_001.pdf]

Supporting Information

Bubble rupture & bursting velocity of complex fluids

Nicola Antonio Di Spirito[1], Shadi Mirzaagha[1], Ernesto Di Maio[1], Rossana Pasquino[1,*], & Nino Grizzuti[1]

[1] *Università degli Studi di Napoli Federico II, DICMaPI, P.le Tecchio 80, 80125 Napoli, Italy*

**corresponding author: r.pasquino@unina.it*

Figure S1 describes the reproducibility of three measurements with the same inflation flow rate for the 0.10 wt% Carbopol solution. The figure reports the measurements of r(t) at Q = 1062 mm^3/s.

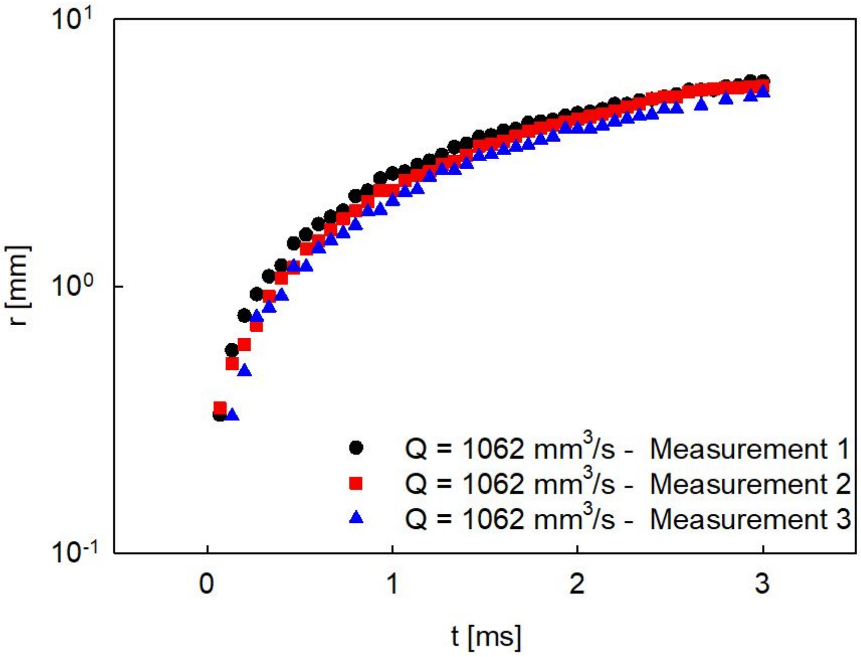

Figure S1. Comparison of three measurements of r(t) at Q = 1062 mm^3/s for the 0.10 wt% Carbopol solution.
